# Commissioning [Integrated] Care in England: An Analysis of the Current Decision Context

**DOI:** 10.5334/ijic.6693

**Published:** 2022-10-07

**Authors:** Pamela Gongora-Salazar, Margaret Glogowska, Ray Fitzpatrick, Rafael Perera, Apostolos Tsiachristas

**Affiliations:** 1 Health Economics Research Centre, Nuffield Department of Population Health, University of Oxford, Oxford, GB; 2 Nuffield Department of Primary Care Health Sciences, University of Oxford, Oxford, GB; 3 Nuffield Department of Population Health, University of Oxford, Oxford, GB

**Keywords:** commissioning, priority-setting, investment decision, integrated care, new models of care, England

## Abstract

**Background:**

The emergence of Integrated Care Systems (ICSs) across England poses an additional challenge and responsibility for local commissioners to accelerate the implementation of integrated care programmes and improve the overall efficiency across the system. To do this, ICS healthcare commissioners could learn from the experience of the former local commissioning structures and identify areas of improvement in the commissioning process. This study describes the investment decision process in integrated care amid the transition toward ICSs, highlights challenges, and provides recommendations to inform ICSs in their healthcare commissioning role.

**Methods:**

Twenty-six semi-structured interviews were conducted with local commissioners and other relevant stakeholders in South East England in 2021. Interviews were supplemented with literature.

**Results:**

England’s local healthcare commissioning has made the transition towards a new organisational architecture, with some integrated care programmes running, and a dual top-down and bottom-up prioritisation process in place. The commissioning and consequent development of integrated care programmes have been hindered by various barriers, including difficulties in accessing and using information, operational challenges, and resource constraints. Investment decisions have mainly been driven by national directives and budget considerations, with a mixture of subjective and objective approaches. A systematic and data-driven framework could replace this ad-hoc prioritisation of integrated care and contribute to a more rational and transparent commissioning process.

**Conclusion:**

The emerging ICSs seem to open an opportunity for local commissioners to strengthen the commissioning process of integrated care with evidence-based priority-setting approaches similar to the well-established health technology assessment framework at the national level.

## Introduction

Over the last decade, England has introduced multiple reforms and initiatives to promote integration of care in the NHS [1,2]. The 2022 Health and Care Act is the latest and farthest-reaching reform, introducing numerous changes to the local commissioning of healthcare. Clinical Commissioning Groups (CCGs), established in 2012 and responsible for commissioning most of the hospital and community NHS services in their local areas [3,4,5] were replaced by Integrated Care System (ICSs). Each ICS will have an Integrated Care Board, which will take on the NHS planning functions previously held by the CCGs. Within each ICS, Integrated Care Boards, health and social care providers, and the local authorities, who are responsible for commissioning social care services [3], are expected to work under a new statutory partnership to promote integration and deliver the best health and social care to their local populations [5,6,7,8]. For that to happen, each Integrated Care Board and their partner local authorities are being required to establish an Integrated Care Partnership [9]. Some CCGs started to merge and work as non-statutory ICSs since 2020, with ‘*integrated care programmes*’ or ‘*new models of care*’ as the spearhead to promote the integration agenda [10,11,12].

With such a major transformation in the local commissioning of healthcare, there is an increased need for evidence-based decision support for ICSs. The removal of competitive tendering in the procurement of clinical healthcare services, included in the 2022 Health and Care Act, is expected to facilitate integration within the NHS, and consequently, to accelerate the implementation of integrated care programmes [8]. Likewise, local governments are expected to play a more prominent role in ICSs than they did under the CCGs arrangements, offering more opportunities for partnership between health and social care [13,14]. Health and Wellbeing Boards, established under the 2012 Health and Social Care Act as formal committees that bring together local authority and NHS representatives [15], will remain responsible for carrying out a joint needs assessment and developing a joint health and wellbeing strategy for their local communities [9,16]. It, however, remains to be seen how Integrated Care Partnerships and Health and Wellbeing Boards will work together [9]. ICSs are also assuming more functions than the CCGs, with additional budgets under their responsibility [17]. Between 70 and 80% of the NHS resources are being allocated to the ICSs across the country [18,19]. Expectations and demands for integration are higher than ever, and under a highly restrictive budget, local commissioners are urged to increase efficiency, while improving healthcare quality and outcomes [6,12].

However, the local commissioning decisions in England are complex, involve several stakeholders, and are not always drawn on evidence [20,21]. It is argued that local commissioners do not have enough capacity to use all routinely collected data and make well-informed decisions [21,22,23,24]. After nearly a decade, it is still unclear how local healthcare commissioners allocate budgets to healthcare services, particularly in the context of integrated care. It is yet not clear what factors drive the local decision-making process, who is involved, and what are the main challenges that commissioners face when investing in one intervention over others. This is unlike priority-setting at national level, where healthcare spending decisions are routinely informed by economic evaluations conducted by the National Institute for Health and Care Excellence (NICE) [24,25].

This paper aims to describe the investment decision process in integrated care in England amid the transition toward ICSs, highlight the main challenges, and provide recommendations to overcome them. Even though the integration agenda is intended to promote integration within healthcare and between health and social care, this paper focuses on the healthcare commissioning process driven by the CCGs. The commissioning experience from the CCGs, including the identification of potential areas for improvement, may help ICSs to integrate care across different organisations and settings as efficiently as possible, and allocate the NHS budgets to maximise population health, improve patient experience, and decrease health inequalities.

The paper is structured as follows: In the following background section, we describe the former and current local commissioning contexts in England. Afterwards, we describe the methodology followed by this study and then summarise the results in five general topics. We discuss the findings of this research and close the paper with the conclusion section.

### The local commissioning context

#### Before the 2022 Health and Care Act

Healthcare commissioning is a continual multifaceted process that involves understanding of population needs, prioritising and planning the services to meet those needs, purchasing services on a limited budget, and monitoring the provision of the procured services [4]. There are multiple actors involved in this process, and the CCGs were the main actors in the commissioning decisions [26]. These groups were not structured in the same way, but they all had a commissioning directorate composed of lead commissioners, directors and managers of the different teams or work-streams (e.g. Planned care & long-term conditions), clinical leads (e.g. Clinical lead for mental health), and the board and the executive team. The later included representatives of the finance, governance, transformation, equality, clinical and commissioning teams.

CCGs primarily consisted of GPs and were responsible for commissioning GP, community health, acute care and mental health services for their local communities [3]. The money came down in cascade from the Department of Health & Social Care and NHS England to all the CCGs, following allocation formulae [27]. These resources represented 70–80% of the NHS England budget [28,29]. These budgets were tied to specific funding streams determined by NHS (e.g. cardiovascular disease budget, mental health budget), and within these allocations, which were predominately historically set, they funded a wide range of services, including integrated care programmes. Even though there was no dedicated budget to fund all these programmes, the Better Care Fund, a pooled budget of £6.5 billion (2021) between the NHS England and local authorities, was used to plan and implement integrated health and social care services [30,31].

**Figure 1:**
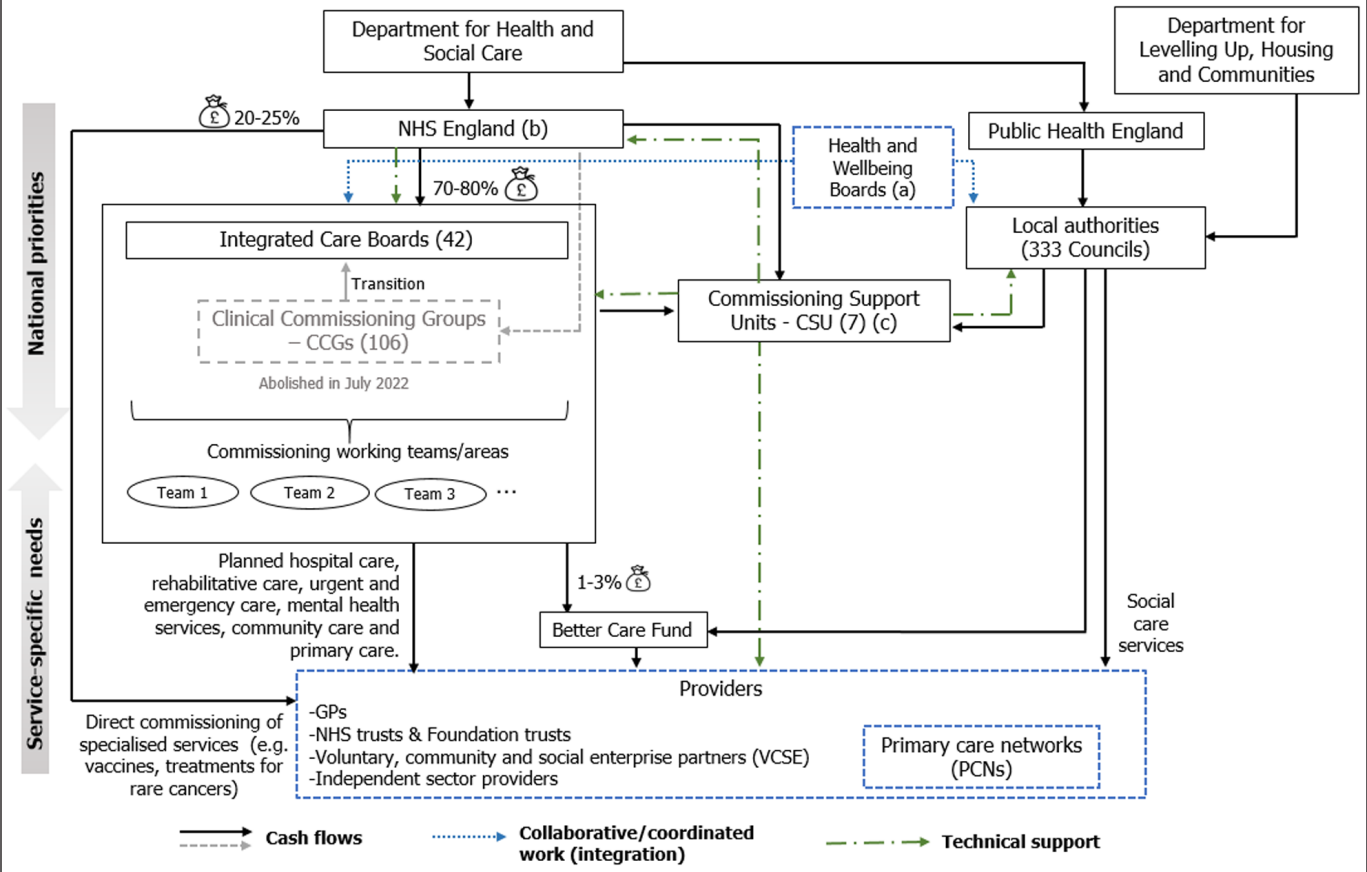
Former commissioning decision-context & cash flow. Percentages (%) in the figure refer to proportion of the total NHS England budget. (a) Some have other joint commissioning structures, such as Health, Education and Social Care, Social Care boards. (b) Since April 2019, NHS England and NHS Improvement work together as a single organisation [33]. (c) NICE, the NIHR and the Academic Health Science Networks could also offer some technical support to CCGs and providers. *Source*: National Audit Office (2018) [34] ; Health and Care Bill (2021) [7]; Health and Social Care Act (2012) [15]; King’s Fund (2020, 2021) [16,28]; Tikkanen et al (2020) [35]; NHS England & NHS Improvement (2021) [19].

Although CCGs made the final investment decisions, they were encouraged to work in coordination with local authorities, mainly via the Health and Wellbeing Boards [32]. The commissioning support units (CSUs), which operate as external consultancy advisory, provided business intelligence, health & clinical procurement, and contract management services to support the CCGs [33]. Figure 1 summarises the former decision context and the main cash flows.

#### After the 2022 Health and Care Act

**Figure 2:**
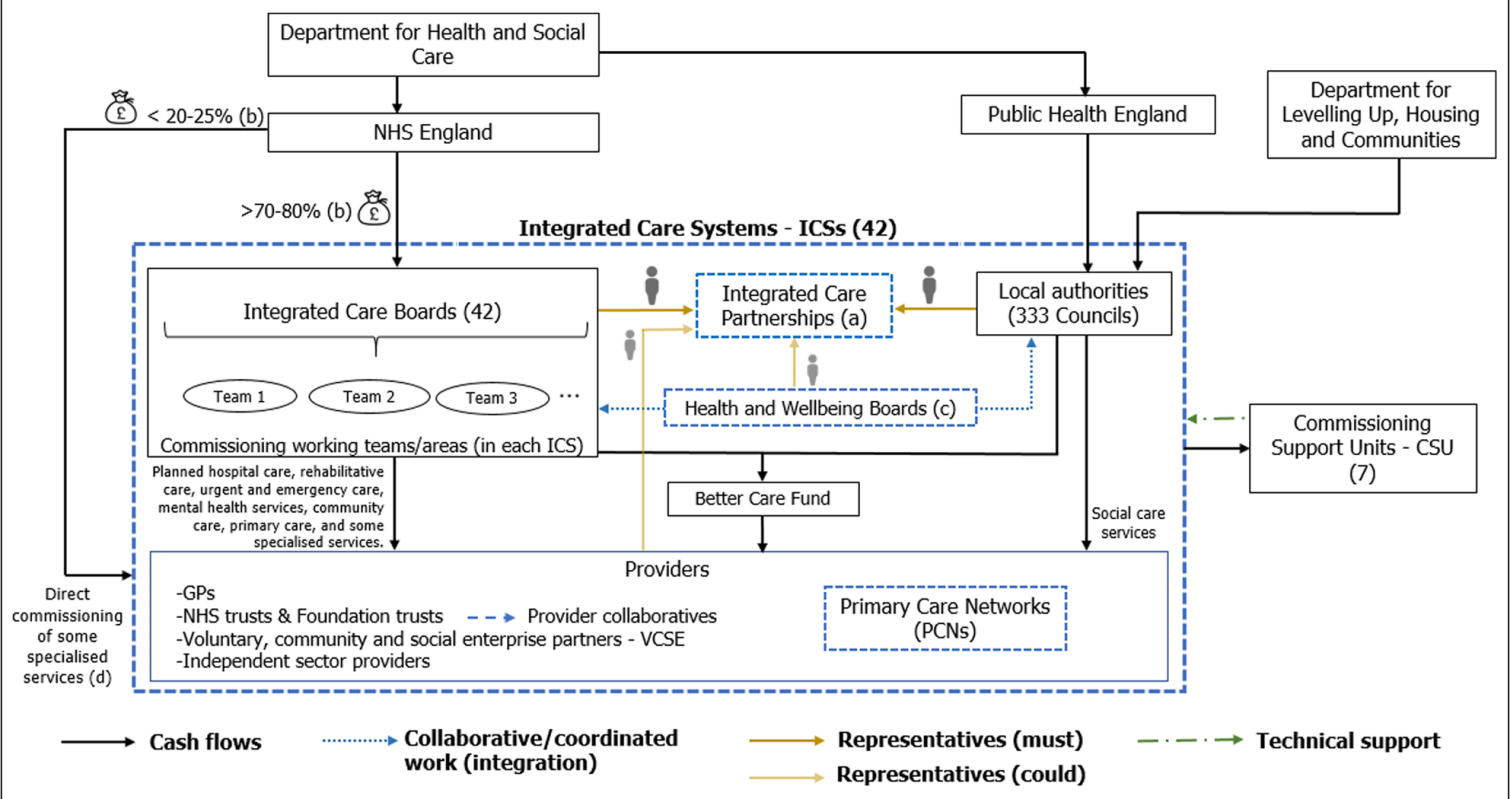
Commissioning decision-context after the abolishment of CCGs. Percentages (%) in the figure refer to the proportion of the NHS England budget. (a) Integrated Care Partnership members must include representatives from the local authorities and from the Integrated Care Boards. Beyond this, members may be from other organisations, including housing and education providers [38]. (b) Financial allocations to each Integrated Care Board will also include resources for a range of functions currently held by NHS England and NHS Improvement (e.g. other primary care budgets) [38]. (c) In some ICSs there might be more than one Health and Wellbeing Board. The Health and Care Bill maintains current arrangements for these boards, but does not define the relationship between them and the ICS partnerships [7,32]. (d) With the 2022 Health and Care Act, Integrated Care Boards are now allowed to “take on delegated responsibility, where appropriate, for commissioning specialised services but within a framework of continued national accountability, national standards, national service specifications and national clinical policies determining equal access to the latest treatments and technologies” [17, p2]. *Source*: The Health and Care Bill (2021) [7], The Health and Care Act (2022) [5], and NHS England guidelines (2021–22) [36,41].

The 2022 Health and Care Act is changing the commissioning architecture. Within each ICS, Integrated Care Boards are assuming the local NHS planning functions and allocation decisions that the CCGs had, and Integrated Care Partnerships are being established as a broad alliance between the NHS, local authorities and providers [5,6,36,37]. Local authorities remain responsible for social care services in the ICS area [38]. Each Integrated Care Partnership is in the process of developing an ‘integrated care strategy’ for its whole population [39]. Providers are becoming constituent members of the Integrated Care Partnership and the Integrated Care Boards. GP practices are being grouped into primary care networks to operate in specific neighbourhoods within the ICS [3]. Along with this, some ‘provider collaboratives’ are emerging as partnership arrangements involving two or more NHS Trusts [40]. Figure 2 summarises the new local commissioning decision context.

ICS arrangements vary significantly, depending on the size and scale of each system. Each ICS is defining its place-based partnership arrangements, with small ICSs operating mainly as a single place-based partnership [42]. NHS England is already making financial allocations to each one of the ICSs [43], and each system is deciding how to distribute these resources across their place-based partnership and the different providers [44]. Integrated Care Boards are responsible for deciding whether allocations are routed through a series of contracts, or whether they prefer delegating the responsibility for securing services for a population [5]. Contracts between Integrated Care Boards and providers may be managed by place-based partnerships or provider collaboratives. This transformation is not entirely new since, in some places, providers already took commissioning powers for specialised mental health services [3]. As was the case with the CCGs, Integrated Care Boards are able to commission jointly with local authorities. More pooled budgets and joint appointments across the two organisations are likely to emerge as a result of this transition [3,38]. Appendix 3 provides a summary of the new local commissioning structures.

## Methods

Semi-structured interviews were conducted with local commissioners and other relevant stakeholders in South East England [45]. The topic guide was designed to identify how is integrated care defined by commissioners, who are involved in the commissioning of integrated care programmes, how these programmes are assessed, how are financial flows structured and how are investment priorities set. The topic guide was tested in two pilot interviews, adjusted accordingly and further refined after the first few interviews to ensure that questions and concepts were not ambiguous. The topic guide is presented in Appendix 1.

A purposive-snowballing sampling approach was adopted to recruit interviewees from two large ICSs located in South East England [45]. Local leaders of commissioning processes were identified through mapping work and suggestions from health experts working at the Oxford Academic Health Science Network and the National Institute for Health Research (NIHR) Oxford and Thames Valley Applied Research Collaboration. Potential participants were selected based on their experience or knowledge of local healthcare commissioning. Roles and type of stakeholders were pre-specified to guarantee equal representation of all levels and seniority. Appendix 2 provides a list of interviewees, their anonymous codes, and their roles.

As a result of the above process, thirty-seven stakeholders were invited by email that included a participant information sheet about the research study, of whom 26 took part in a semi-structured interview (70% response rate). From the interviewees, 6 were directors/chief officers, 5 were chair, accountable officers or executive leads, 4 were head/lead commissioners, 3 were programme managers, 3 were clinicians (pathway leads), 3 were CSU members, and 2 were public health registrars. More than 80% of the interviewees were CCG or Sustainability and transformation partnership (STP) members. All interviews were conducted online via Microsoft Teams 2017 [46] between April and July 2021, and were audio-recorded with the participants’ consent. Interviews were then transcribed verbatim by a professional transcriber and one of the researchers verified the transcriptions against the audio-recordings [47]. NVivo 12 software [48] was used to categorise and analyse data collected. When developing the thematic coding framework a hybrid approach of inductive and deductive coding was adopted [49], with themes being identified based on analysis of raw data and the four main interview topics (see Appendix 1). A selection of interview excerpts are shown in quotation marks, although supplementary quotes are provided in Appendix 4.

Findings from the interviews were supplemented with existing evidence from the literature, and were used to contribute to the discussion section. Relevant studies were identified through searching Google and Google Scholar in July 2021 and using a combination of the following search terms: “commissioning”, “priority-setting”, “investment”, “healthcare”, “integrated care”, “new models of care”, “local”, and “England”. Reports published by independent organisations in the UK that focus on integrated care and commissioning, such as the King’s Fund, were also considered.

Ethics approval was granted by the University of Oxford’s Medical Sciences Interdivisional Research Ethics Committee (Reference: R74765/RE001).

## Results

Five general topics were identified based on the analysis of the interviews:

### Understanding of integrated care initiatives

When interviewees were asked to define integrated care, most of them provided a person-centred definition, highlighting the importance of adopting a holistic approach when addressing individuals’ needs, and consequently the relevance of services working together regardless of the organisation the teams or care professionals belong to.

“it’s literally everybody in all aspects working together to identify what the challenges are, what it is we need to overcome, and how we work together” (No. 8)

There are multiple integrated care programmes in place, although there is not a formal terminology to refer to these initiatives (e.g. programmes, interventions, work-streams). Participants distinguished between two types of integrated care programmes that in practice tend to overlap: (i) condition-specific interventions with elements of integration, such as the ‘Integrated diabetes care programme’, and (ii) interventions that look at the wider picture and overlap with the condition-specific programmes, such as the ‘Aging well programme’, which looks at individuals with 3 or 4 long-term conditions. Examples of integrated care programmes in place, mentioned by the participants, are provided in Appendix 4.

Despite the existence of these programmes, some of them are not yet fully integrated in practice. According to some interviewees, most of the integration has arisen in the form of partnerships, via memorandums of understanding, rather than in the provision of care sphere. Patients’ information is not yet shared with all teams involved, limiting the coordination in the planning and provision of care. Among other limitations, participants argued that the lack of flexibility in the contractual structure precludes the reorganisation of care services to operate under a common structure. Some participants were also sceptical about the idea of integration, arguing that it is an over-used phrase or an unfilled promise made 20 years ago.

### Investment decision process

According to the interviewees, there was a top-down and bottom-up prioritization process within the CCGs. On the one hand, every year NHS England set national priorities and provided guidance to the CCGs, indicating the outcomes to be achieved. Based on this, CCGs tailored local patient pathways and introduced changes in the provision of care. Some arrangements were also directly introduced at national level, irrespective of what was happening locally, or what was seen as priorities for the CCG governing body. Some examples are the introduction of the remote ‘total triage’ model in 2020 [50] or the six new models of care outlined in the 2014 Five Year Forward View [51]. Based on this, the CCG governing body or the Health and Wellbeing Boards discussed and set priorities, which were subsequently communicated to the different commissioning working teams [39,52].

**Figure 3:**
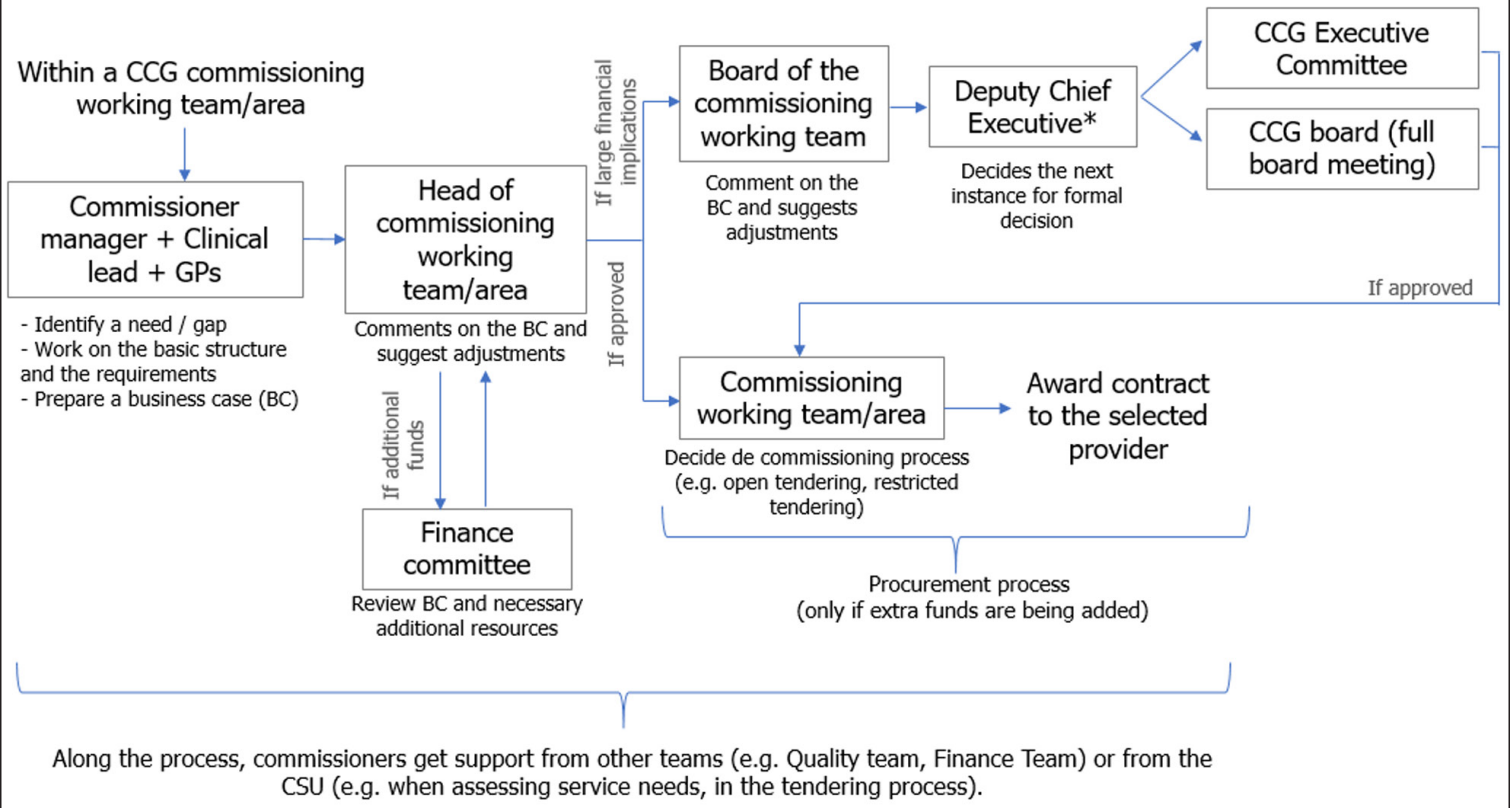
Bottom-up investment decision process within a CCG. * When explaining this process, some participants referred to the ICS while others to the CCG role. *Source*: Based on interviews conducted between April and July 2021.

On the other hand, priorities also emerged from needs or gaps that commissioners identified locally. Each commissioning working team worked together with GPs and clinical leads to identify improvements needed. If a service-specific need was detected, the commissioning working team worked on the basic structure and requirements of that service, and prepared a business case. Managers costed up the proposed improvement and checked whether it was following all the equality and diversity legislation [53]. The finance team took part in the approval decision if the proposal required additional funds. If the new service or arrangement in the proposed business case had a substantial investment implication, the board of the corresponding commissioning working team reviewed the business case. Subsequently, the business plan was examined by the Deputy Chief Executive for approval, who then decided whether to send it to the CCG Executive Committee or to the CCG board for formal decision (see Figure 3).

The NHS England was normally not involved in the local commissioning of services, unless the proposal had a direct or indirect impact on specialised services. If the business case involved arrangements around the provision of social care or the use of funds from pooled budgets between the council and the CCG, public health teams from the council also got involved in the shaping and implementation of the business case. Although patients and the public were sometimes involved in the commissioning process, it seems that there was a lack of clear arrangement for making their engagement more systematic.

“For patient involvement, that depends very much on the project manager in the area of work. Some are very good at involving patients and making sure there’s an input and engagement, and others not” (No. 18)

Participants did not mention major differences between the commissioning of individual interventions and integrated care programmes.

Despite the new management structures and committees, in the opinion of various interviewees, most of the CCG staff will be transferred to the Integrated Care Boards. This goes in line with NHS guidance, indicating that each CCG’s staff, assets and liabilities will be transferred to the relevant Integrated Care Board [41]. No substantial changes were expected in the commissioning process, as many features of CCG were believed to remain within the new place-based partnership arrangements. By the time the interviews were conducted, however, there was a great deal of uncertainty on how each of the ICSs will develop and how the organisational landscape will look like.

“So I’d say the main difference would be that we would have providers in the conversations much earlier than we used to and sitting alongside us” (No. 23)

### Prioritisation of interventions

Participants were asked about the approaches taken by the CCG to prioritise health interventions, including integrated care programmes, and the factors that drive local investment decisions. Various participants maintained that there was no structured framework but rather a mixture of subjective and objective approaches, with no differences between simple or single interventions and integrated care programmes. The many objectives to fulfil and the multiple sources of information to account for, hindered the development of a robust prioritization process using explicit criteria. Some interviewees, however, highlighted the existence of some frameworks or reports, such as the Joint Strategic Needs Assessment [39] or the products developed by the RightCare Intelligence Programme [54] which allowed them to identify local needs or quality issues. In spite of this, decisions were not always guided by evidence. Almost all of the participants argued that NHS England directives and budgetary considerations were the main drivers of prioritization of health interventions at local level. Although to a minor extent, some participants also mentioned leadership and ability to lobby for particular interventions as potential determinants of final investment decisions.

“I think we are heavily influenced by national directives […] And the second thing […] I would say the finance, I’m afraid, does influence it big time” (No. 20)

These investment drivers contrast with the criteria that, according to the respondents, should be guiding the local investment decision process. Table 1 provides an overview of these ideal criteria and the frequency of interviewees that mentioned them. Health outcomes at individual and population level were highlighted by over 90% of the interviewees that addressed this question. Similarly, quality of care, including patient experience, was underlined by the majority (70%). The third most popular criteria was the ‘cost/financial considerations’. These first criteria are known in the literature as the ‘Triple aim’ of integrated care [55,56,57], a framework directly mentioned by some of the participants.

**Table 1:** Ideal criteria for prioritization of interventions.


CRITERIA	# PARTICIPANTS

**Health outcomes** e.g. *‘mortality and morbidity assessments’(No.5) ‘QALYs’(No.17), ‘HbA1c’ (No.2)*	24

**Quality of care** e.g. ‘*Best possible health and care experience’ (No. 1), ‘better access, like reducing waiting times’ (No. 16); ‘‘getting the pathway right’(No.22)*	18

**Cost/budget considerations** e.g. ‘*financial implications of the programmes’(No.14); ‘financial burden of the condition’(No.2); ‘saving money’(No.15)*	12

**Efficiency** e.g. *‘value for money’(No.12); ‘cost effectiveness of that service’(No.25)*	7

**Equity** e.g. ‘*how well resources are distributed to different groups in the population’ (No.10); ‘reduce unwarranted variation’(No.3); ‘equality’(No.24)*	6

**Compliance to policies/priorities/guidance** e.g. ‘*national directives’ (No.20), ‘integration with social care services’ (No.1)*;	5

**Size of the population** e.g. ‘*make a difference to a large amount of people’(No.15); ‘high prevalence conditions’ (No.12)*	3

**Others** e.g. *‘meeting a completely unmet need’(No.18); ‘whole system benefit’(No.26); ‘focuses more on prevention’(No.8)*	


*Note*: In total, the 26 participants responded to this question.

### Assessment of interventions

The assessment of healthcare interventions, including integrated care programmes, did not seem to be a systematic process and varied across interventions. For certain specialities, there were some population health monitoring tools in place, mostly in the form of dashboards, with developments at national and CCG levels [58]. Some one-off evaluations were also conducted, using a mixture of qualitative and quantitative methods, aiming at identifying service failures or assessing the efficiency or effectiveness of interventions. Most of these evaluations, however, apparently lacked methodological rigorousness and there were only few counterfactual analyses [59].

“It hasn’t been systematic. It’s been driven by looking at the integrated care program that we put in place, trying to work through what we think the impact has been, uhm… and using data to back that up. The problem has been demonstrating cause and effect” (No. 6)

To compare healthcare interventions against standards, national trends, or other regions, participants mentioned multiple national [54,60,61] and local [39,62] sources of information. Examples of these are the clinical audits and registries run by NHS Digital [61] and the CCG Integrated Performance Report [62], which looks at the overall performance of the CCG and the associated organisations. These tools, however, do not include in detail information of interventions’ performance, but rather a summary of performance and quality issues across broad areas within the CCG. Finance, quality and service issues with current providers used to be reviewed based on key performance indicators (KPIs). Despite the multiplicity of indicators, no metric has been developed to measure the degree or success of integrated care, and few KPIs can be used for comparison purposes. This is particularly relevant when health inequalities is one of the major NHS England concerns [12].

Prospective evaluations were also mentioned by participants, and were normally included in the business cases that working teams prepared. Some CCGs conducted quality and equity impact assessments, in which they considered the potential impact of the new programme or service on patient safety, clinical outcomes, patient experience of care delivery, and equality of access.

### Challenges in the commissioning process

Access and use of information was one of the most common barrier highlighted by interviewees. Relevant data was sometimes held by different organisations, with different IT systems, making it difficult for commissioners to access timeliness linked databases. In some cases, commissioners had to pay or obtain permission to use certain databases (e.g. NHS primary care). Participants also mentioned data quality issues and information overload, which prevented commissioners from conducting a comprehensive analysis of local population needs [63]. The need to gather more data on patient experience or satisfaction was also highlighted [55]. Some interviewees also argued that CCGs lacked the capacity to use all data available to inform the commission process. The CSUs offered support in this regard, although various participants saw them as bureaucratic and unresponsive organisations. This might partially explain why CCGs bought fewer services from CSUs over the last years while investing in developing more in-house capabilities [34].

“I do think it’s access to information. I think it’s an access to data and being able to pull the data and being able to understand the data” (No. 1)

Financial constraints were also underlined as a challenge by most interviewees. The combination of increasing demand for care and costs, achieving new targets, and dealing with reduced funding, made it difficult to finance integrated care. An increasing number of CCGs were overspending against their planned expenditure [34]. Furthermore, each one of the commissioning working teams had to work within the funding allocation to different services set by the centre [19,29], which limited CCGs’ ability to redirect funds and reprocess investment.

Operational challenges were also highlighted. Most of the contracts with providers were long and had different termination dates, hindering the planning and implementation of service transformations. The procurement of services itself was also stressed as a barrier due to the limited staff, the complexity of all associated rules and policies, and with this, the involvement of many actors. Workforce constraints not only impaired the procurement process but all other commissioning tasks. Recruiting and retaining skilled health and social care workers was constantly a challenge, particularly in rural areas. Inequalities in access to healthcare due to limited supply of services or quality issues in under-served areas of the country have been documented in the literature [64,65,66,67,68,69] and acknowledged by the NHS England [12,70].

From a governance point of view, nearly all participants considered that CCGs had little flexibility to set local priorities. According to some interviewees, the pressure to comply with the multiplicity of NHS England constitutional targets, together with the constant changes introduced at national level, obstructed the prioritisation of local needs. The lack of a truly integrated system approach was also stressed by various participants. Despite all organisational changes introduced over recent years, the different teams and organisations worked in silos, with competing financial priorities and a focus on acute care. In some cases, there was also a disconnection between the executive committee and those on the ground doing the commissioning. In this context, working relationships played a crucial role in the planning and delivering of integrated care.

All these financial and human resources limitations, together with contractual and procurement constraints, hindered the commissioning of integrated care. It seems that most of the commissioning was about introducing changes around the edges, rather than radical modifications to the way services are being provided to the population.

As expected, the COVID-19 pandemic had an enormous effect on the commissioning of healthcare. The local commissioning process stiffened due to an increasing ‘command and control’ from the centre, and the shift to remote work affected to an extent communication flows. This also affected the implementation of integrated care programmes in itself, as face-to-face meetings can be crucial in developing trust and relationships between actors involved [71]. Interesting enough, COVID-19 also played a ‘catalyst role’, as the pandemic forced many of the CCG teams to work closer. Some information and governance structures were developed to ease the COVID-19 response, and some of these apparently extended to other healthcare conditions.

According to some interviewees, the Health and Care reform might have the potential to address some of the commissioning challenges mentioned above [6,8]. Some participants indicated that ICSs were starting to invest in new software developments and analytics [36,72] to aggregate and analyse data from multiple care providers. Others also mentioned that changes to the system architecture are expected to offer commissioners more flexibility in terms of budget allocations and the use of their workforce across larger geography, with the possibility to gain some efficiency. Under the new procurement system, the role of competition and tendering is being reduced, and local commissioners are allegedly having more flexibility in the arrangement of services [38].

Some respondents were however more sceptical, and saw this transformation as a top-down reorganisation of the commission system. According to some of them, ICSs will not solve the problem of providers being financially constrained to operate on a system basis. Bringing all stakeholders together will probably be the biggest challenge, particularly for ICSs merging with big or multiple CCGs. Others also suggested that national priorities will still dominate over the local ones. Nevertheless, by the time the interviews were conducted, there was still uncertainty around the statutory framework, and the NHS was in the process of publishing guidance on the implementation of ICSs [36].

## Discussion

The local healthcare commissioning context in England is transitioning towards a new organisational architecture, aiming to strengthen the NHS integration agenda. Although there was a broad consensus among interviewees on the benefits of integrated care [73], most integrated care programmes were still in the process of implementation and their commissioning is yet to be finalised. This is possibly due to the various barriers in the commissioning process identified in our study.

ICSs are building on the CCG legacy and inheriting most local commissioning processes and structures that the CCGs developed. In light of this and based on our findings, we provide recommendations to ICS commissioners on potential ways to overcome the commissioning barriers identified.

The reported need to improve data availability and quality [21,26,71,74,75] is of paramount importance for the successful development of integrated care programmes and the realization of their expected benefits. Routine collection and use of data related to the triple aim, particularly on patient experience, would enable the monitoring and performance evaluation of integrated care programmes. Although CSUs were partly conceived to support commissioners with the access and use of data, there was a general preference –among commissioners- for CSUs services to be carried out directly by the CCGs or ICSs. This is in line with the findings of Petsoulas et al (2014) [75]. It might be therefore necessary to redefine the role of CSUs under the new commissioning landscape, although, so far, none of the NHS England guidelines [36] mentions how are CSUs expected to support ICSs.

In regards to the reported difficulties in patient and public involvement [PPI] in the commissioning process, with the emerging ICSs such difficulties should be resolved by defining a clear plan on where, how, and to what capacity PPI will be part of the process [11,26,76]. A formalized space for PPI within the ICS structure is probably necessary for PPI to be a reality in the local commissioning process [77], and Integrated Care Boards have legal duties in this regard [3,5,78].

Moreover, our findings also highlighted the need to strengthen the evaluation culture and capacity, and ensure that evaluations are effectively embedded in the local commissioning process. Robust evaluations, particularly of integrated care programmes, have proved to be not only beneficial but also highly valued by commissioners and providers [71,79]. Even though current evidence indicates that integrated care is likely to reduce cost and improve outcomes [including health and patient experience] [80,81,82], more rigorous economic evaluations are needed as the evidence is of poor methodological quality [82,83]. In evaluating integrated care programmes, it is essential to acknowledge the complex nature of these interventions and the consequent limitation of traditional analytical methods [56,57,84,85,86]. Multi-criteria decision analysis, for instance, has been suggested as a sound and comprehensive method that can overcome the limitations of traditional frameworks in the context of integrated care [87,88,89].

Although it is unclear how the top-down and bottom-up prioritization processes interacted, national directives and financial constraints played a predominant role in local healthcare investment decisions. This contrasts with the multiplicity of reports and data sources available. These results are in line with findings from Currie et al [2018], who concluded that commissioners had insufficient capacity to use evidence to inform decisions and powerful actors may influence knowledge absorption processes in the CCGs, aiming to achieve their goals [21]. The disconnection between evidence and final investment decisions can be partially explained by the absence of a structured prioritisation framework. The local commissioning of healthcare operates under a complex and interactive system that hardly follows a rigid orderly logic [20,90], and the priority-setting process inevitably involves social value judgements and political tensions [23,90,91]. Therefore, a value-based framework is desirable for efficiency, transparency and accountability. Our findings revealed what seems to be a set of desirable prioritisation criteria, with the triple aim framework at the front. This criteria set could be used by local commissioners to develop a clear prioritisation framework that acknowledges the complexity of integrated care programmes.

To develop such a framework, a good reference point could be the health technology assessment framework established at national level to guide priority-setting decisions. Under this framework, NICE provides economic evaluations routinely to advise NHS England and NHS Improvement on reimbursement decisions of new medical technologies [92]. More than 1,130 individual appraisal recommendations have been published since 2000, mainly based on cost-effectiveness analysis [93,94]. For over 20 years NICE has forged a great reputation for a robust and transparent appraisal of the best available evidence, and is today one of the oldest and most successful organisations worldwide in using economic evidence to improve efficiency in healthcare [25,93]. Similarly, NICE or a new governmental agency could provide a framework for commissioning decisions at local level.

With the ICSs now in place, it is also worth reflecting on the distinction between the commissioning of simple interventions and integrated care programmes. In contrast with simple interventions, integrated care requires, for instance, the involvement of multiple actors and joint or more flexible contracting tools [24]. Simple interventions are, however, progressively disappearing as multidisciplinary teams and integrated care pathways are becoming part of usual practice through updated guidelines [95,96,97]. This might explain why interviewees did not raise differences between the commissioning of simple interventions and integrated care. In light of this, it is desirable to promote commissioning processes and tools, including contractual models, suitable for integrated care.

Although it is yet to be seen how the 2022 Health and Care Act will address all challenges in the commissioning of integrated, our findings suggest that the majority of commissioners perceived the ICSs as an opportunity to pool budgets, implement shared-decision making and materialize the idea of integrated care. This enthusiasm was also identified by the King’s Fund when exploring how ICSs are starting to develop across the country [11]. It is however too early to reach conclusions as there is still uncertainty on many administrative and operative aspects of the new structures [11,16,71].

It is expected that this study encourages an open discussion within the emerging ICSs on the barriers and enablers of the local commissioning of integrated care. Various participants mentioned that the questions we asked were pertinent, and that the issues identified should be widely discussed and addressed collectively within the emerging ICSs. We also believe that findings of this study are likely to be of international interest as countries are gradually moving towards integrated care [57,98], and local purchasers of healthcare from high-income economies are likely to encounter similar challenges as the ones faced by local commissioners in England.

### Strengths and Limitations

One of the major strengths of the study is the diversity of the 26 interviews conducted, with professionals with different roles and varying levels of seniority. The plurality of interviewees allowed us to build a more comprehensive view of the commissioning challenges and bring commissioners’ voices at all levels to the ongoing discussion on how to improve the commissioning of integrated care. Conducting this research in the midst of the transition toward ICSs is the second strength. This increases the chances that results are taken into consideration by local commissioners, as they are currently in the process of defining how the new commissioning structures will work in practice, and how they will relate to one another. Results of this study have been already presented to the local commissioners involved in the interviews, and are currently being used for discussions within some of the ICS’ teams. The novelty of this study is the third strength as this is the first study that explores how local commissioners in England invest in integrated care, to the best of our knowledge. Previous studies have focussed on the use of evidence in the commissioning process [20], the prioritisation of public health interventions [23], the development of integrated care programmes [11,71], the contractual options for integrated care [24,99] or the effectiveness of clinical leadership in the commissioning of healthcare [26].

The external validity of these findings is limited by the focus of this study on only two ICSs in South East England. Future research would benefit from the inclusion of more ICSs, as there is great variation in terms of the size, complexity and development of the different ICSs across the country [11]. Nevertheless, the two ICSs selected for the study have different governance structures and are at a different level of maturity. These differences, together with the coincidences that we found with previous literature, make our findings likely to be transferable to other ICSs. Another limitation arises from the lack of interviewees from NHS England or the Department of Health and Social Care despite all our efforts. Their perceptions would have enriched this study with their perspective and views, as they are the main precursors of the transformation in the local commissioning process. Similarly, we interviewed only two public registers from the local authorities and did not examine the local commissioning of social care in depth. Exploring the commissioning process from the local authorities’ perspective would have offered a more comprehensive understanding of the local commissioning of integrated care. Nevertheless, it remains to be seen how Integrated Care Partnerships and Health and Wellbeing Boards will work together. Furthermore, one could argue that the transitional nature of the context could make obsolete some of the findings of this study in the near future. However, the CCGs’ staff is being transferred to the corresponding Integrated Care Board [41], and in the opinion of various interviewees, no substantial changes were expected in the commissioning process.

### Conclusion

The new local commissioning structures offer an opportunity for commissioners to strengthen and rationalise the commissioning process of integrated care. Improvements in collecting and using performance data and adopting an evidence-based priority-setting framework could contribute to overcoming the challenges faced by the CCGs and improving local investment decisions in England. To make progress in this regard, commissioners can learn from the experience accumulated by the well-established health technology assessment framework at the national level.

## Data Accessibility Statement

Due to ethical concerns, the interview transcripts cannot be made openly available.

## Additional File

The additional file for this article can be found as follows:

10.5334/ijic.6693.s1 Appendix – Supplemental material. Appendices 1 to 4.
