# Supplementary material for: Commissioning [Integrated] Care in England: An Analysis of the Current Decision Context
Source: Int J Integr Care. 2022 Oct 7;22(4):3. doi: 10.5334/ijic.6693 (PMC9541117; doi:10.5334/ijic.6693)
Supplement: Appendix – Supplemental material. — Appendices 1 to 4. [file ijic-22-4-6693-s1.pdf]

Appendix - Supplemental material

Appendix 1. *Semi-structured interview guide*

1. GENERAL BACKGROUND

1. Can you tell me a bit about your role? Which team are you part of? And, for how long have you been in the CCG/STP/ICS?
2. Are you involved in the decision-making process around integrated care programmes?

2. INTEGRATED CARE PROGRAMMES

1. Could you tell me which integrated care programmes (or programmes with elements of integration) are in place at the moment in the CCG/STP/ICS, and how do they work?
2. Who set the budget across health programmes within the CCG? How are financial flows determined and influenced? Is there a specific budget for integration?
3. How would you define integrated care programmes?

3. ASSESSMENT OF HEALTH INTERVENTIONS

1. In the context of integrated care programmes, how are health interventions assessed?
2. What kind of data or information is normally used for that purpose, and where does the data come from?
3. What would you say are the main constraints or challenges that the CCG/STP/ICS face when assessing health interventions?

4. COMMISSIONING

1. Who are generally involved when commissioning integrated care programmes? Any organisation from outside?
2. How would you describe the approaches taken in this local authority to develop priorities for integrated care spending or disinvestment? Is there any decision-making tool in place? What kind of information is used to inform prioritisation decisions?
3. What sorts of factors influence commissioners to invest in one integrated care programme over others? How is this going to change in the light of the transition?
4. What do you see as the main barriers that the CCG/STP/ICS face when making commissioning decisions of integrated care programmes?

Appendix 2. *List of interviewees*

Participant No.	Role
1	Programme manager – CCG/STP
2	Pathway lead - CCG/STP
3	Executive lead - CCG/STP
4	Pathway lead - CCG/STP
5	Accountable officer - CCG/STP
6	Director - CCG
7	Programme manager – CCG
8	Head/lead commissioner – CCG
9	Public health registrar - County Council
10	Programme manager – CCG
11	Chair/Executive lead – CCG
12	Director – CCG
13	Chief officer - CCG
14	Director – CCG
15	CSU member
16	Director – CCG/STP
17	Former Chair/Executive lead – CCG
18	Head/lead commissioner – CCG
19	CSU member
20	Chair/Executive lead – CCG/STP
21	Chair/Executive lead - CCG
22	Head/lead commissioner - CCG
23	Head/lead commissioner – CCG/STP
24	Director - CCG
25	Public health registrar - County Council
26	CSU member

Appendix 3. Local structures under the new commissioning context

Structure	Definition
Integrated Care Board	"A new type of NHS body that bring the NHS together with partners across their area. Integrated care boards are responsible for developing a plan for meeting the health needs of the population, managing the NHS budget, and arranging for the provision of NHS services in their area" (1)
Integrate Care Partnership	"Each integrated care board and their partner local authorities will be required to establish an integrated care partnership, bringing together health, social care, public health (and potentially representatives from the wider public space where appropriate). Integrated care partnerships will be tasked with promoting partnership arrangements to address the health, social care and public health needs of their system" (1)
Health and Wellbeing Board	"A committee of the partner local authority, often considered to be at 'place' level (although 'places' are locally determined, this will not be the case in all areas). Established by the Health and Social Care Act 2012 they must include listed representatives from the local authority, local Healthwatch, and each

	integrated care board in the local authority's area, alongside such other persons as the local authority considers appropriate. They produce a joint strategic needs assessment and a joint local health and wellbeing strategy."[1]
Place-based partnerships	"Collaborative arrangements formed by the organisations responsible for arranging and delivering health and care services in a locality or community. Place-based partnerships will remain as the foundations of integrated care systems as they are put on a statutory footing (subject to legislation), building on existing local arrangements and relationships" [2]
Provider collaboratives	"Provider collaboratives are partnership arrangements involving at least two trusts working at scale across multiple places, with a shared purpose and effective decision-making arrangements, to:<br>• reduce unwarranted variation and inequality in health outcomes, access to services and experience<br>• improve resilience by, for example, providing mutual aid<br>• ensure that specialisation and consolidation occur where this will provide better outcomes and value.<br>Provider collaboratives work across a range of programmes and represent just one way that providers collaborate to plan, deliver and transform services. Collaboratives may support the work of other collaborations including clinical networks, Cancer Alliances and clinical support service networks." [3]

1. Department of Health & Social Care. Annex B: glossary. Definitions of terms found in 'Guidance on the preparation of integrated care strategies' 2022 [cited August 2, 2022; updated July 29, 2022]. Available from: https://www.gov.uk/government/publications/guidance-on-the-preparation-of-integrated-care-strategies/annex-b-glossary

2. NHS England and NHS Improvement. Thriving places. Guidance on the development of place-based partnerships as part of statutory integrated care systems. London, UK; 2021 September 2, 2021. [cited August 2, 2022]. Available from: https://www.england.nhs.uk/wp-content/uploads/2021/06/B0660-ics-implementation-guidance-on-thriving-places.pdf

3. NHS England and Improvement. Working together at scale: guidance on provider collaboratives. 2021 August, 2021. Report No.: PAR754. [cited August 2, 2022]. Available from: https://www.england.nhs.uk/wp-content/uploads/2021/06/B0754-working-together-at-scale-guidance-on-provider-collaboratives.pdf

Appendix 4. *Supplementary quotes and information provided by interviewees*

Understanding of integrated care initiatives

Table 1. *Examples of integrated care programmes in place in two ICSs from South East England, mentioned by interviewees*

Condition-specific programmes	Programmes across conditions
Integrated diabetes care programme (No.1)	*Aging Well programme (No. 1)*
Cardiovascular disease programme (No.16)	*Enhanced Health in Care Homes (No. 1)*
Musculoskeletal programme (No. 22)	*Urgent Community Response (No.11)*
Ophthalmology programme (No. 8)	*Primary Care Transformation Programme (No.13)*

- *"you know, you don't refer to all them as programmes, formally speaking, but rather you, you use another terminology, other name to refer to all them. Yeah. It'll be, it'll be, it'll be, it'll be 'projects', 'programmes', 'workstreams' (No. 2)*
- *"[...] I think of it sometimes because, you know, we've got very different organisations with all their own individual cultures, objectives, all the rest of it. And they, it, it, you know, the reality of integration is that you've got your own governance processes within those organisations, so you have to ... (noise) Sorry, that's the front door, you have to, you know, you have to meet the requirements of your own individual organisations as, which means that integration quite often means additional work rather than, it's an 'as well as' rather than an 'instead of'.(No. 24)*
- *"But I would, I would say that probably most of the, most of the integration work is on a more of a partnership basis, through things like memorandums of understanding and joint working, rather than vertical integration end of the sphere." (No. 23)*
- *"I think that has been limited to date, in my view, by the combination of a lack of, of real understanding of need, of, you know, of, of population data and also the, the, the, the, the great difficulty that systems and senior managers have responding to this because of the combination of the, you know, what, the, the cultural challenges of, of people being locked into roles, you know, where this is what we do, I do this, blah blah blah, and the, the central demands that come down all the time, you know, you've got to launch this service or you've got to do that. So I think it, it is very challenging although, you know, there are starting to be some evidence of success" (No. 17)*

Investment decision process

- *"So, how it normally works is that the commissioners, like the colleagues you've already spoken to at the CCG, they will identify a need for a service, and they will, will sort of work on the, the basic structure and the requirements of that service in the service spec" (No.12)*
- *"R: So how much flexibility would you say the CCG has to, to set local priorities?*

I: Very little really. It's very centrally driven and finance driven. So although we spend a lot of time, you know, trying to behave as commissioners and coming up with good ideas, you have to demonstrate that the, the main demonstration is that it essentially saves money or that it will save money, not that it's necessarily the best thing to do." (No. 21)

- *"But I think in terms of the decision making, I mean, you would need to go through the CCG decision-making processes about something like this. So say you were gonna commission a new service, for instance, or you were gonna fundamentally change the way you commission something, and particularly if it has a financial implication, what you would, what you would do is, is, you would do a, a report for the, the Executive Committee. I mean, you, you would do a report generally. So I would, in my case, for instance, I would normally take that, you know, I'd, I'd work up a report or a briefing around this, and a proposal, if you like, with, including any, kind of, financial and contractual implications. Now, I would, in the first instance, sort of, work that up and take that, you know, through to, you know, like, the head of Planned Care, who's my boss, and take, you know, for comment. And, and then take it through to, say, the, the Planned Care Programme Board within the CCG in the first instances. After that, you know, including, kind of, you know, any comments around, around that, I'd then, sort of, take it through to our director for, for our, for us, which is the, the, the Deputy Chief Executive of the CCG, who we would take that to. And then she will take a view on it and, you know, and, you know, and decide whether it should go through, it will go through to the Executive Committee for a formal, kind of, decision, or, you know, and, you know, to maybe get advice on this from the Director of Governance as to whether it needs to go through to a formal decision making at the, the CCG board, the full board meeting" (No. 10)*
- *"I don't think it'll change hugely, but I think that's also to be determined, because somebody's still gonna have to make decisions about how the money is spent" (No. 16)*
- *"so whilst we're awaiting legislation under the white paper, we are working on the assumption that we as an integrated care system will receive the allocation for the total population of [name of ICS]. And that will sum to £[.] billion, there or there about. And then we're trying to work through how we will, how we will allocate that resource and whether that allocation will be routed through a series of contracts for the provision of services. Or whether we will be delegating the responsibility for securing services for a population through to different sub-ICS structures like local authority pooled budgets, or provider collaboratives, or an alternative set of arrangements. So that's the bit of design we're going on at the moment." (No.5)*
- *"Although until now, until the merge of the CCG came into effect all the governance of decision-making was in the local CCG's. So, very little was done centrally." (No. 6)*

Prioritisation of interventions

- *"National priorities, areas of local needs, that kind of thing. So areas of, you know, that have been identified as areas of local need. Or quite often like if there's a problem, it, it, it, there, if there's a problem with a service, although that wouldn't be investing in one over and above another, it would just be focusing on one." (No. 24)*
- *"I would say, and it is cynically, there are a number of drivers for the areas of work we develop. There are, there are very clear national drivers established from central government [...] There are other things that come in because we are, we know there are challenges, failures within our existing system [...] There are other things that then start to get more interest, pressure based, personal interest based, which, sort of, come from a wide number of sources. So, you might have a particular clinician that's really keen on doing one thing, and has good networks, good ways of communicating that, good ways of on-boarding people to their, to their issue. And that develops, sort of, a, a momentum of itself. So, there are individual, individuals who, who are championing a cause in a very powerful way that that gets done" (No. 7)*

Assessment of interventions

- *"there's a number of ways that we would do that within the CCG. So you've got, you've got something like, for instance, the Integrated Performance Report [...] you will, there'll, there'll also be, you know, certain metrics and things that will be gathered from things like the Quality and Outcomes framework in Primary Care [...] So, and you'll also have things like the [place] Joint Strategic Needs assessment [...]" (No. 10)*
- *"So, there'd be hard metrics of, of, of, you know, patient metrics of money, attendances, is one way. And that's, you know, and then performance against constitutional standards, which we always have to look at. But then there would be patient reported outcome measures, and patient confidence measures." (No. 11)*
- *"[...] the differential impact of Covid by ethnicity over the past 12 months has highlighted the importance of data and we know that some of our data, in terms of impact and burden of disease, either at very high resolution within different parts of the county or for different potentially more marginalised in one of the population groups, isn't as good as it should be." (No. 25)*
- *"So the traditional commissioning assessment of things is through I guess three main possible routes, the, the top route usually being, "Is it costing us more than we can afford? Which is a very familiar problem and one that CCGs are obsessed about for the length of their existence. The other one is a quality perspective, so are we seeing the kind of outcomes we would expect for patient care? Are we seeing serious events that we would want to prevent? That kind of stuff. And the third one is probably general performance, so for example around cancer care, we work closely with the services delivering cancer care, particularly looking down to tumour site, add (?)*

waiting times and the processes in place within providing organisations to make sure waiting times are not any longer than they absolutely have to." (No. 18)

- *"It will vary by programme. So, if they're … so, I don't, I don't think we have any, well in fact I know, we don't have kind of KPIs related to "integration" and what integration means and how you might measure that and how you might understand success with that. So, that isn't … doesn't happen. But then of the programmes that we might class as being integrated, then they will have with their own individual KPIs in terms of process and outcome measures." (No. 25)*

Challenges in the commissioning process

- "[...] *So I, I think again, I mean CSUs are used because CCGs have to use them […] we need a, a better system. It needs to be, they need to be owned by the integrated care systems not some external consultancy advisory which just peddles the same stuff and sells it five times to different people, different CCGs" (No. 17)*
- *"You, you know, we need to start commissioning by people's needs, rather than, than services. I suppose some of the restrictions to that are the, the length of contracts that we have for different providers who provide these services. They don't always end up the, same time, so, you know, we, we, we'll renew a contract, but what it doesn't, what we don't take into consideration is actually, "Right, these, these people need this bundle of care, and what, what contracts are we going to, to consolidate and integrate to, to achieve that? We're, we're, we don't tend to end contracts early. We don't tend to, to do a lot of procurement, to, to achieve that." (No. 12)*
- *"Historic focus on organisational targets can be distracting from taking a step back and prioritising a … prioritising something. The procurement rules are very constraining and really don't encourage people to do … make many changes, if any. And the third thing that's a constraint is the … the complexity of delivering an integrated out … care pathway outcome. It demands so many facets that you can't easily evaluate the integrated component. You can measure activity by organisation, but … but we don't have joined up data sharing yet. So, it … to do any integrated pathway at the moment relies on every organisation sharing their component part of an integrated pathway. Joined up data would enable that to be more easily done, so that we measure the outcome by the person's experience of the pathway, rather than by the organisation's intervention. (No. 20)*
- *"the money side of things is obviously a massive issue, because they have got so many things that come down from NHS England that they are meant to achieve, and, you know, and, and the funding just isn't there, and at the same time, they also know what their local population needs, and there may not be funding available to do that. So, you know, that's a, that's the, that's one of the big things. And, and having the right staff is quite often an issue, you know? There's … I've been on projects over the years in the NHS where it has surprised me about how little has been*

achieved because it's decisions by committee, rather than just getting on and achieving something" (No. 15)

- *"I think the other thing I've not mentioned that is an important driver and one that's deeply frustrating is because of the financial regime we work within, everything has to deliver within a year which is not realistic." (No. 18)*
- *"And we started to make progress, some areas more than others. But then the pandemic hit, and it really meant we, we had to quite rightly pause everything, as a lot of services got stood down." (No. 8)*
- *"Well, I think obviously there's always a finite financial envelope, isn't there, there's always, you know, there's not an unlimited pot of money that we can spend and I think we all acknowledge, you know, the, the impact of Covid and how, you know, the impact that that, the expenditure on Covid is going to have on, on the NHS and what it might mean for future budgets and just generally, you know, the, the, the amount that the government spends on the health service can't just keep going up and up and up and up, you know, there are limits on what we can spent." (No. 2)*
- *"Yeah. We are working much closer with one another. The information constraints and also the governance structures around Covid are going to help that as well. So, the different boards, the Health Protection Board, part of ... sort of the local silver and bronze structures, etcetera." (No. 25)*
- *"The transition from CCGs to integrated care systems is creating more of an opportunity for providers to collaborate together and to together take a responsibility for their population, rather than to assume it's the CCG's responsibility." (No. 20)*
- *"Well, I think on paper it should definitely help. Whether it, whether it will I think remains to be seen. But the fact that there's less different organisations should help. There's only the one CCG that eventually, I think, provide commissioners sit around one table, on paper looks like it should help with those sort of conversations." (No. 3)*
- *"I still think it's early days and it's not clear yet what will be done centrally at scale and what will still be left to local decision. I think I think probably that that's the big challenge for next year is is to describe that narrative about how you run programs at scale, while still having local accountability and local decision-making. This is one of those things there's not a right answer. You've got to have the local you've got to have the at-scale, the Community program I'm involved in is a really good case in point. You know so…we work as as a group of commissioners across [locations' name] footprint defining care pathways, doing evaluation impacts… uhm but the conversations about how we move the money or support are still local conversations, because of course you've got local trusts are the building blocks to this, and they're the ones which are feeling the impact. So, I think there's a real opportunity… I think that the ICS Financial*

framework, which is being developed, will be key to this, because It's all about governance; If we get the governance right and get the right decisions taken at the right point." (No. 6)
